# The Genetic and Molecular Basis of O-Antigenic Diversity in *Burkholderia pseudomallei* Lipopolysaccharide

**DOI:** 10.1371/journal.pntd.0001453

**Published:** 2012-01-03

**Authors:** Apichai Tuanyok, Joshua K. Stone, Mark Mayo, Mirjam Kaestli, Jeffrey Gruendike, Shalamar Georgia, Stephanie Warrington, Travis Mullins, Christopher J. Allender, David M. Wagner, Narisara Chantratita, Sharon J. Peacock, Bart J. Currie, Paul Keim

**Affiliations:** 1 Department of Biological Sciences, Northern Arizona University, Flagstaff, Arizona, United States of America; 2 Menzies School of Health Research and Northern Territory Clinical School, Darwin, Australia; 3 Faculty of Tropical Medicine, Mahidol University, Bangkok, Thailand; 4 The Translational Genomics Research Institute, Flagstaff, Arizona, United States of America; Yale University School of Medicine, United States of America

## Abstract

Lipopolysaccharide (LPS) is one of the most important virulence and antigenic components of *Burkholderia pseudomallei*, the causative agent of melioidosis. LPS diversity in *B. pseudomallei* has been described as typical, atypical or rough, based upon banding patterns on SDS-PAGE. Here, we studied the genetic and molecular basis of these phenotypic differences. Bioinformatics was used to determine the diversity of genes known or predicted to be involved in biosynthesis of the O-antigenic moiety of LPS in *B. pseudomallei* and its near-relative species. Multiplex-PCR assays were developed to target diversity of the O-antigen biosynthesis gene patterns or LPS genotypes in *B. pseudomallei* populations. We found that the typical LPS genotype (LPS genotype A) was highly prevalent in strains from Thailand and other countries in Southeast Asia, whereas the atypical LPS genotype (LPS genotype B) was most often detected in Australian strains (∼13.8%). In addition, we report a novel LPS ladder pattern, a derivative of the atypical LPS phenotype, associated with an uncommon O-antigen biosynthesis gene cluster that is found in only a small *B. pseudomallei* sub-population. This new LPS group was designated as genotype B2. We also report natural mutations in the O-antigen biosynthesis genes that potentially cause the rough LPS phenotype. We postulate that the diversity of LPS may correlate with differential immunopathogenicity and virulence among *B. pseudomallei* strains.

## Author Summary

*Burkholderia pseudomallei* is an environmental Gram-negative bacterium and the cause of melioidosis, an often life-threatening disease affecting people in Southeast Asia and northern Australia. Melioidosis is usually contracted by bacterial inoculation, ingestion or inhalation. Effective vaccines for melioidosis are currently unavailable. This organism contains a large genome, which varies greatly among strains due to a high frequency of genetic recombination. We report here on diversity of lipopolysaccharides (LPS) in this species, a major component of the bacterial outer membrane and a known immunogenic virulence factor. We developed LPS genotyping techniques to study frequency of two major LPS types, known as typical and atypical LPS, in *B. pseudomallei* strains collected from two endemic regions: Southeast Asia and Northern Australia. LPS genotype variation differed among *B. pseudomallei* populations. During the investigation, we discovered a new LPS genotype in a sub-population group of *B. pseudomallei* in Australia. We postulate that such differences are likely to be associated with variable immunopathogenicity and clinical presentation in the human host.

## Introduction

Lipopolysaccharide (LPS) is a major component of the outer membrane of Gram-negative bacteria, playing an important role in cell integrity and in signaling host innate immune response [1]. Structurally, LPS is composed of three major components: lipid A, the bacterial endotoxin that is embedded in the phospholipid bilayer of the outer membrane; core-oligosaccharide; and O-antigen. These three components are linked together as a part of the bacterial outer membrane. In a highly pathogenic bacterial species, such as *Burkholderia pseudomallei*, LPS has a major role in stimulating host innate immune response during infection [2]. *B. pseudomallei* LPS has been classified as a type II O-polysaccharide (O-PS) and is one of 4 different surface polysaccharides produced by this pathogen [3]. Previous studies have shown that *B. pseudomallei* LPS is required for serum resistance and virulence [4]. It has been well established in many bacterial diseases that overstimulation of the host cells by LPS can lead to the features of septic shock [5]. Likewise for *B. pseudomallei*, septicemia is a major cause of death. In Northeast Thailand especially in Ubon Ratchathani Province where melioidosis is highly endemic, the average incidence rate of melioidosis is 12.7 cases per 100,000 people per year with the average of 42.6% of mortality rate [6]. Cellular recognition of LPS by the innate immune system triggers the proinflammatory cytokines by host cells, which aids in the clearance of the pathogen. Previous studies have supported a potential role for *B. pseudomallei* LPS in protective immunity, with high concentrations of antibodies to LPS associated with survival in severe melioidosis [7], [8]. As a result, LPS has been used in vaccine development and provided protective immunity in a murine model of melioidosis [2]. In addition, it was demonstrated that LPS had an important role in bacterial virulence because the LPS mutant *B. pseudomallei* strain SRM117, which lacked the O-antigenic polysaccharide moiety was more susceptible to macrophage killing during the early phase of infection than its parental strain 1026b [9].

A previous study [10] identified LPS diversity based upon electrophoretic mobility with SDS-PAGE and detection using immunoblot analysis. This diversity included two serotypes (A and B) possessing different electrophoretic ladder profiles and a rough type that did not contain the ladder patterns; all were antigenically distinct [10]. Molecular structure of O-antigen serotype A or typical type has been described as the unbranched heteropolymers consisting of disaccharides repeats of -3)-β-D-glucopyranose-(1-3)-6-deoxy-α-L-talopyranose-(1- in which approx. 33% of the L-6dTal*p* residues bear 2-*O*-methyl and 4-*O*-acetyl substituents whereas the other L-6dTal*p* residues carry only 2-*O*-acetyl substituents [11]. We note that the structures are not known for any of the other *B. pseudomallei* O-antigens. *B. thailandensis*, a genetically related non-pathogenic species, has LPS that is cross-reactive to sera obtained from *B. pseudomallei* and *B. mallei* infections, and this has led to the development of a vaccine for melioidosis using LPS from *B. thailandensis* [12]. *B. mallei*, the causative agent of glanders, has O-antigen structure similar to those found in *B. pseudomallei* and *B. thailandensis*, except that it has different side-group modifications at the L-6dTal*p* residues which lack the acetylation at the *O*-4 position [13]. These structural differences are associated with the absence of *oacA* gene in *B. mallei*. *oacA* encodes for O-antigen acetylase A in *B. thailandensis* and its homolog in *B. pseudomallei* K96243 is identified as BPSL1936 [14].

*B. pseudomallei* genomes are very diverse due to horizontal gene transfer events [15], [16] and dynamic changes in repeated sequences [17]. This results in diverse phenotypic characteristics such as bacterial colony morphotypes [18], and importantly, may be implicated in the diverse clinical manifestations observed among melioidosis patients. The latter range from asymptomatic cases, to localized infections, to whole body sepsis, along with differential seroreactivities [19], all of which may be correlated with the great genomic diversity in this species [15], [17]. Nevertheless, the specific roles of genetic diversity in *B. pseudomallei* in differential clinical presentations of melioidosis requires further analysis, as clinical studies suggest host risk factors are the major determinant of disease severity [20]. Because LPS phenotypic diversity is important for serology and diagnostics, we investigated the genetic and molecular basis of differential LPS phenotypes in a large *B. pseudomallei* population. Bioinformatics, phenotypic characterization, as well as, population genetics approach were used in this study to better understand this important trait.

## Methods

### Comparative genomics of LPS biosynthesis genes

Artemis and Artemis Comparison Tool (ACT) software [21] was used to display and compare multiple *B. pseudomallei* genomes. Genomes and nucleotide sequences used in this study are listed in Table 1. Mutations in O-antigen biosynthesis genes were identified using basic homologous gene based alignments.

**Table 1:** LPS genotype identification and mutations in the four closely related *Burkholderia* species.

Species	Strain	Source	GenBank Accession Number	LPS Genotype	*oacA*
*B. pseudomallei*	K96243	Human	NC_006350	A	Intact
*B. pseudomallei*	1026b	Human	AF064070	A	Intact
*B. pseudomallei*	1106a	Human	NC_009076	A	Intact
*B. pseudomallei*	1106b	Human	NZ_CM000774	A	Intact
*B. pseudomallei*	1710a	Human	NZ_CM000832	A	Intact
*B. pseudomallei*	1710b	Human	NC_007434	A	Intact
*B. pseudomallei*	MSHR668	Human	NC_009074	A	Intact
*B. pseudomallei*	MSHR1655	Human	NZ_CH899712	A*	Frame-shift
*B. pseudomallei*	MSHR305	Human	NZ_AAYX01000005	A	Intact
*B. pseudomallei*	MSHR346	Human	NC_012695	A	Intact
*B. pseudomallei*	MSHR840	Human	GU574442	B2	Intact
*B. pseudomallei*	MSHR139	Human	HM852063	B2	Intact
*B. pseudomallei*	MSHR1950	Human	HM852062	B2	Intact
*B. pseudomallei*	112	Human	NZ_ABBP01000549	A	Frame-shift
*B. pseudomallei*	14	Animal	NZ_ABBJ01000798	A^†^	Intact
*B. pseudomallei*	406e	Human	NZ_CH899732	A	Intact
*B. pseudomallei*	576	Human	NZ_ACCE01000003	B	Intact
*B. pseudomallei*	7894	Human	NZ_ABBO01000695	A	Intact
*B. pseudomallei*	9	Human	NZ_ABBL01000749	A	Intact
*B. pseudomallei*	91	Animal	NZ_ABBK01000735	A	Intact
*B. pseudomallei*	B7210	Human	NZ_ABBN01000620	A^‡^	Intact
*B. pseudomallei*	BCC215	Human	NZ_ABBR01000422	A	Intact
*B. pseudomallei*	DM98	Human	NZ_ABBI01002075	A	Intact
*B. pseudomallei*	NCTC13177	Human	NZ_ABBQ01000469	B	Intact
*B. pseudomallei*	Pakistan 9	Human	NZ_ACKA01000012	A	Intact
*B. pseudomallei*	Pasteur 52237	Human	NZ_CH899755	A	Intact
*B. pseudomallei*	S13	Human	NZ_CH899770	A	Intact
*B. mallei*	ATCC23344	Human	NC_006348	A	Absent
*B. mallei*	NCTC10229	Animal	NC_008836	A	Absent
*B. mallei*	NCTC10247	Unknown	NC_009080	A	Absent
*B. mallei*	SAVP1	Animal	NC_008785	A	Absent
*B. mallei*	2002721280	Unknown	NZ_CH899691	A	Absent
*B. mallei*	ATCC10399	Animal	NZ_CH899681	A	Absent
*B. mallei*	FMH	Human	NZ_DS264097	A	Absent
*B. mallei*	GB horse4	Animal	NZ_AAHO01000001	A	Absent
*B. mallei*	JHU	Human	NZ_DS264109	A	Absent
*B. mallei*	PRL-20	Animal	NZ_AAZP01000025	A	Absent
*B. thailandensis*	E264	Environment	NC_007651	A	Intact
*B. thailandensis*	Bt4	Environment	NZ_ABBH01000548	A	Intact
*B. thailandensis*	TXDOH	Human	NZ_ABBD01000533	A^‡^	5′truncation
*B. oklahomensis*	C6786	Human	NZ_ABBG01000257, NZ_ABBG01000258	N/A	Intact
*B. oklahomensis*	EO147	Human	NZ_ABBF01000376	A^‡^, ^ψ^	Intact

Note: LPS genotypes and mutations were identified in the main O-antigen biosynthesis gene locus and the *oacA* homologs. Frame-shifted mutations of the O-antigen biosynthesis genes were found in their *wbiI*(*), *wbiF*(^†^),*wbiE* (^‡^), and *wbiD* (^ψ^) genes. We did not test the effects of *wbiF* mutant in *B. pseudomallei* 14, and *wbiE* mutant in *B. pseudomallei* B7210, due to unavailability of live bacterial cultures. The listed GenBank accession numbers are associated with the LPS genotype identification, not for *oacA* analysis. Details of the *oacA* mutations are demonstrated in Figure S1.

### PCR Analysis

**Figure 1:**
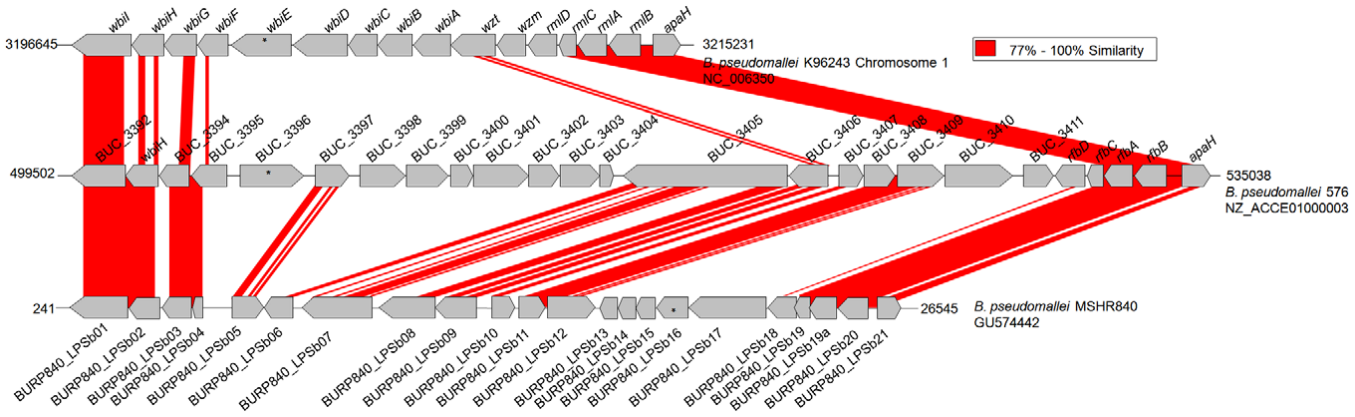
Diversity of three biosynthesis gene clusters for the O-antigen moiety of the LPS in *B. pseudomallei*. The typical LPS ladder pattern (i.e serotype A) was associated with O-antigen biosynthesis gene cluster in K96243 (top) or LPS genotype A, whereas the atypical LPS ladder pattern (serotype B) found in approximately 13.8% of Australian strains was believed to be associated with a different O-antigen biosynthesis gene cluster or LPS genotype B observed in strain 576 (middle). Strain MSHR840 was identified as a variant serotype B strain, designated as LPS genotype B2, because many of its O-antigen biosynthesis genes (bottom) were similar to those found in strain 576. We note that genes encoding for key components of the O-antigens (e.g., *wbiGHI*, and *rmlBAC*), were conserved across these 3 different clusters. **Note**: * Target genes selected for PCR assays to represent each LPS genotype; GenBank accession number and nucleotide coordinates are indicated for each genome used in the analysis; gene *apaH* is shown in this figure as a flanking gene that is not involved in the O-antigen biosynthesis.

**Figure 2:**
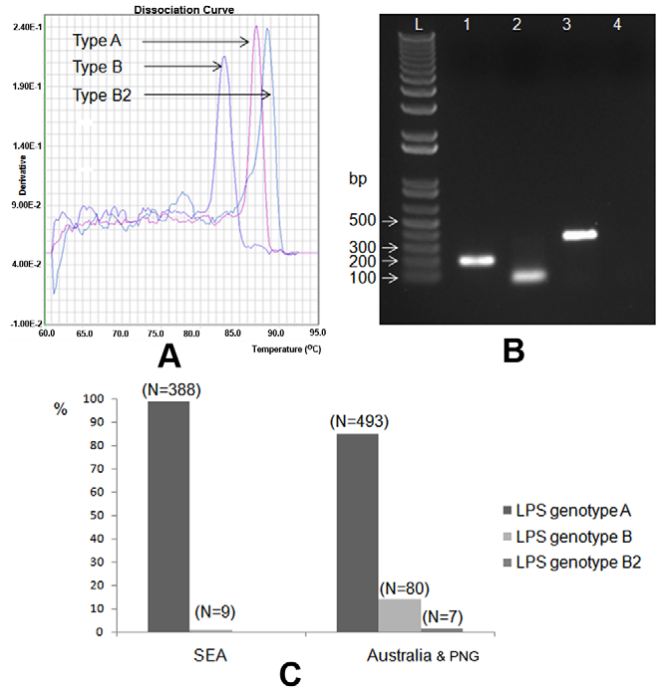
Genotyping scheme and frequencies of three different LPS genotypes identified in *B. pseudomallei* populations. Multiplex SYBR-Green PCR assays were developed to target the presence of genes: *wbiE*, BUC_3396, and BURP840_LPSb16, which were the representatives of LPS genotypes A, B, and B2, respectively. PCR amplicons from these 3 gene targets were differentiated by melting dissociation (A); or sizing (B); lanes 1, 2, 3, and 4 are PCR products from strains K96243, 576, MSHR840, and non-DNA template control (NTC), respectively; and L, 1 kb-plus DNA ladder. We note that LPS genotype A was the most common LPS genotype, whereas a majority of the LPS genotype B was found in strains from Australia (approx.13.8%). Genotype B2 was found in strains from Australia and Papua New Guinea (PNG) only (C).

Multiplex-SYBR-Green PCR assays were designed to target 3 different LPS genotypes. Gene *wbiE* of *B. pseudomallei* K96243, gene BUC_3396 of *B. pseudomallei* 576, and gene BURP840_LPSb16 of *B. pseudomallei* MSHR840 were used as the PCR markers to investigate frequency of LPS genotypes A, B, and B2, respectively (Figures 1&2). PCR primers used in this study are as follows: wbiE_F, 5′-TCAAACCTATCCGCGTGTCGAAGT-3′; wbiE_R, 5′-TCGTCGTCAAGAAATCCCAGCCAT-3′; BUC3396_ F, 5′-AATCTTTTTCTGATTCCGTCC-3′; BUC3396_R, 5′ -ACCAGAAGACAAGGAGAAAGGCCA-3′; BURP840_LPSb16_F, 5′-AACCGGGTAGTTCGCGATTAC-3′; and BURP840_LPSb16_R, 5′-ATACGCCGGTGTAGAACAGTA-3′. The PCR assay was conducted in 10-µL reaction mixtures containing 1× SYBR-Green master mix (Applied Biosystems, USA), 0.3 µM of each PCR primer, and 0.1 to 1.0 ng of DNA template. Most tested DNA samples were made in collaborative laboratories in Thailand and Australia using various DNA extraction techniques. The reactions were performed on an ABI 7900HT Sequence Detection System (Applied Biosystems, USA) utilizing 40 cycles. Each cycle contained two steps: denaturation at 95°C for 15 s and annealing at 60°C for 30 s. The PCR products were further analyzed by melting them continuously from 60°C to 95°C to generate a dissociation curve. The melting temperatures of PCR amplicons for genes *wbiE*, BUC_3396, and BURP840_LPSb16 were constant at 87.0°C, 83°C, and 88.5°C, respectively. We used this assay to analyze DNA templates from 999 diverse *B. pseudomallei* strains isolated from clinical, animal, and environmental samples from Australia (*n* = 600), Thailand (*n* = 349), Malaysia (*n* = 27), Vietnam (*n* = 7), Papua New Guinea (*n* = 2), and unknown countries in Southeast Asia (*n* = 14), as well as 77 *B. thailandensis* strains, 2 *B. thailandensis*-like spp. strains, and 37 strains of unknown soil bacteria.

### DNA sequencing and analysis

Whole genome sequencing was performed using 454 sequencing technology (Roche, USA) by US Army Edgewood Chemical Biological Center (ECBC), MD, USA. Artemis –based analysis and BLAST were used to annotate the O-antigen biosynthesis genes of *B. pseudomallei* strains MSHR840, MSHR139, and MSHR1950. DNA sequencing for *wbiI* and *oacA* genes was performed using ABI 3130×l Genetic Analyzer (Applied Biosystems, USA).

**LPS identification and characterization:** Techniques for LPS extraction and SDS-PAGE analysis followed a previous study [10]. Immunoblot analysis was performed using sera from melioidosis patients with known infection with *B. pseudomallei* LPS genotype A or B strains as the primary antibodies. Horse radish peroxidase (HRP) – conjugated anti-human IgG was used as the secondary antibody in a standard immunoblot analysis. Monoclonal antibody 3D11, the *B. mallei* LPS-specific mAb (Research Diagnostics Inc., USA), was used as a primary antibody in the immunoblot analysis of the *oacA* mutant strains.

### Serum susceptibility tests

Select *B. pseudomallei* strains were tested for growth, multiplication, and survival in the presence of 30% normal human serum (NHS) as previously described [4] with some modifications. Briefly, each *B. pseudomallei* strain was inoculated in a 2 mL of TSBDC media and incubated overnight at 37°C and 250 rpm in an orbital shaker. The overnight culture (100 µL) was used to inoculate 3 mL of TSBDC media and then incubated at the same conditions for 4 hr to reach mid exponential growth phase. Serum susceptibility tests were performed in 1.5 mL microfuge tubes containing 100 µL of bacterial culture, 300 µL of NHS (Lonza Inc., USA), and 600 µL PBS. The mixture was incubated at 37°C for 2 hr, and then the number of viable bacterial cells was determined using plate counting. *B. pseudomallei* 1026b and *E. coli* HB101 were used as positive and negative controls in this study, respectively.

### GenBank Accessions

Nucleotide sequences and annotations of the O-antigen biosynthesis genes in *B. pseudomallei* strains MSHR840, MSHR139, and MSHR1950LPS were submitted to GenBank under accession nos. GU574442, HM852063, and HM852062, respectively.

## Results

To better understand the diversity of genes responsible for the biosynthesis of O-antigen moiety of the LPS in *B. pseudomallei*, we first used a comparative analysis of all publicly available *B. pseudomallei* genomes to identify differences within LPS biosynthetic genes. Three different O-antigen biosynthesis gene categories, or genotypes, were identified. Secondly, we examined the genotype frequencies in *B. pseudomallei* populations using PCR assays targeting each of these genetic types. Thirdly, we correlated LPS genotypes with their differential phenotypes (serotypes). This led to our discovery of a natural mutation in an O-antigen biosynthesis gene in a clonal panel of *B. pseudomallei* strains isolated from a single human host. The adaptability of *B. pseudomallei* strains through LPS variation, even within a single human host, represents an important aspect of pathogen biology and a complication for melioidosis host response.

### Diversity of O-antigen biosynthesis genes in *B. pseudomallei* and its near-relative species

We compared 27 *B. pseudomallei*, 10 *B. mallei*, 3 *B. thailandensis*, and 2 *B. oklahomensis* genomes (Table 1) to identify the LPS O-antigen biosynthesis genes. Assuming synteny and common genomic locations, along with known or predicted function, *B. pseudomallei* O-antigen biosynthesis genes were assigned to two major groups. Group A (LPS genotype A) was identical or very similar to the O-antigen biosynthesis operon observed in *B. pseudomallei* 1026b [4], whereas group B (LPS genotype B) was found in an atypical LPS strain 576 and also in the species type strain, NCTC13177. LPS genotype A was found in most *B. pseudomallei* and all *B. mallei* and *B. thailandensis* genomes examined. Surprisingly, the more distantly related *B. oklahomensis* strain EO147 also had LPS genotype A, which was different from the predicted O-antigen biosynthesis gene cluster in other *B. oklahomensis* strains (C6786, C7532, and C7533; data not shown). This may represent a lateral gene transfer event into EO147 and is deserving of additional study. Furthermore, regions within the two clusters had different levels of sequence conservation. Genes located at the ends of these two clusters (e.g., *wbiGHI*, and *rmlBAC*; Figure 1) had higher sequence similarity than most of the genes in the core of the clusters. Indeed, many of the cluster cores contain distinct gene composition. The conserved genes include those important for oligosaccharide synthesis and O-antigen biosynthesis [4].

LPS genotype frequencies were analyzed across a large strain collection using PCR-based assays. Multiplex-SYBR-Green PCR assays were designed to target a specific gene unique for each genotype. Gene *wbiE* (BPSL2676) of *B. pseudomallei* strain K96243 and gene BUC_3396 of strain 576 were used to represent the presence of LPS genotypes A and B, respectively (Figures 1&2). A total of 999 *B. pseudomallei* strains from different geographic locations and epidemiological origins (e.g., clinical, animal, and environmental strains) were tested for their LPS genotypes. We noted that 23 *B. pseudomallei* strains were collected from one melioidosis patient. We found that LPS genotype A was the most common genotype in both Australian and Southeast Asian strain populations (Figure 2). LPS genotype B was relatively rare in Southeast Asian strains (∼2.3%), but was found in 13.8% of Australian strains. Five strains from Australia and two strains from Papua New Guinea were non-typeable using these two PCR gene markers. Three of these strains, MSHR840, MSHR1950, and MSHR139 were further analyzed for O-antigen biosynthesis gene identification using whole genome sequencing. The O-antigen biosynthesis gene clusters from these strains were identified and annotated (GenBank accession nos. GU574442, HM852062, HM852063). Comparative genomics demonstrated that many genes in this new cluster were similar to those of the LPS genotype B genes of *B. pseudomallei* 576 and were distinct from the K96243 LPS genotype A genes. Hence, these newly identified O-antigen biosynthesis gene clusters represent a variant of the LPS genotype B and, consequentially, were designated as LPS genotype B2 (Table 1). Figure 1 shows the genomic comparison of these three different O-antigen biosynthesis gene clusters: A, B, and B2 (from *B. pseudomallei* strains K96243, 576, and MSHR840, respectively). We note that %G+C content of the core of these 3 different clusters is relatively low (∼59–60%) compared to the conserved parts of the O-antigen biosynthesis operon (∼68%). This supports the hypothesis that these genomic differences are due to genetic recombination e.g., horizontal gene transfer, which is common in *B. pseudomallei* [15], [16]. Comparative genomics of these three different clusters using homologous-based alignment are summarized in Table S1. Again, we note that genes *wbiGHI*, and *rmlBAC* are conserved among these three different clusters. Furthermore, gene BURP840_LPSb16 from strain MSHR840 was selected for use as a PCR marker to represent the LPS genotype B2. PCR genotype analysis (Figure 2) revealed that all seven of the previously non-typeable strains were positive for the LPS genotype B2. The LPS B2 genotype was found only in strains from Australia and Papua New Guinea. It is important to note that there is no known clonal relationship among these seven strains. The LPS B2 genotype genes were also found in a *B. thailandensis*-like spp. strain MSMB121, which was isolated in Australia (unpublished data). Complete LPS genotypic data are reported in Table S2.

### A novel LPS electrophoretic pattern - a type B variant

**Figure 3:**
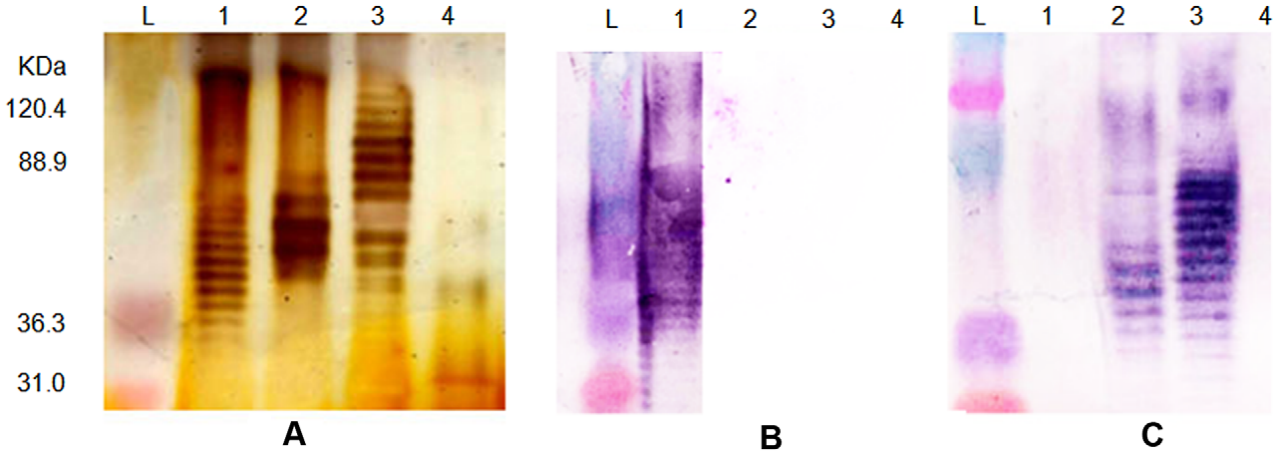
Diversity of *B. pseudomallei* LPS banding patterns and their serological specificity. Panel A is silver strained SDS-PAGE of four different LPS phenotypes; panels B and C are immunoblotting analysis of the same LPS samples using sera from melioidosis patients with known infection by LPS genotype A, or B strains, respectively. Lanes 1–4 are typical (type A), atypical (type B), a novel atypical (type B variant or type B2), and rough LPS types, respectively; lane L is a pre-stained protein standard ladder. We note that the typical LPS was specifically seroreactive to the antibody from patient who was infected by LPS genotype A strain, whereas, the atypical LPS types (lanes 2 and 3) were seroreactive with the antibody from the LPS genotype B infected patient only. Rough LPS or no-banding LPS appearance (lane 4) was seronegative to both sera.

LPS genotyping results were further examined by direct comparison to LPS electrophoretic phenotypes [10]. Due to the difficulty of international Select Agent transfer and BSL3 handling, we phenotyped only ∼ 24% of the isolates that were genotyped. We note that this is a limitation of our study. That said, all examined LPS A or B phenotypes were perfectly matched with their LPS A or B genotypes. In addition, 22 strains producing the rough LPS phenotype were all identified as LPS genotype A (Table S2). The genetic basis of the rough phenotype and its derivation from the A phenotype is known for only 16 of these strains (see below). SDS-PAGE revealed that LPS genotype B2 strains produced a distinct ladder pattern, though they were all detectable with type B sera using immunoblot hybridization. The B2 phenotype had a wider range of molecular weights (40–120 kDa) than the LPS types A and B. In total, three LPS banding patterns plus the rough LPS type (no ladder) can be detected (Figure 3).

### Natural mutations in O-antigen genes and changes in bacterial phenotypes

**Figure 4:**
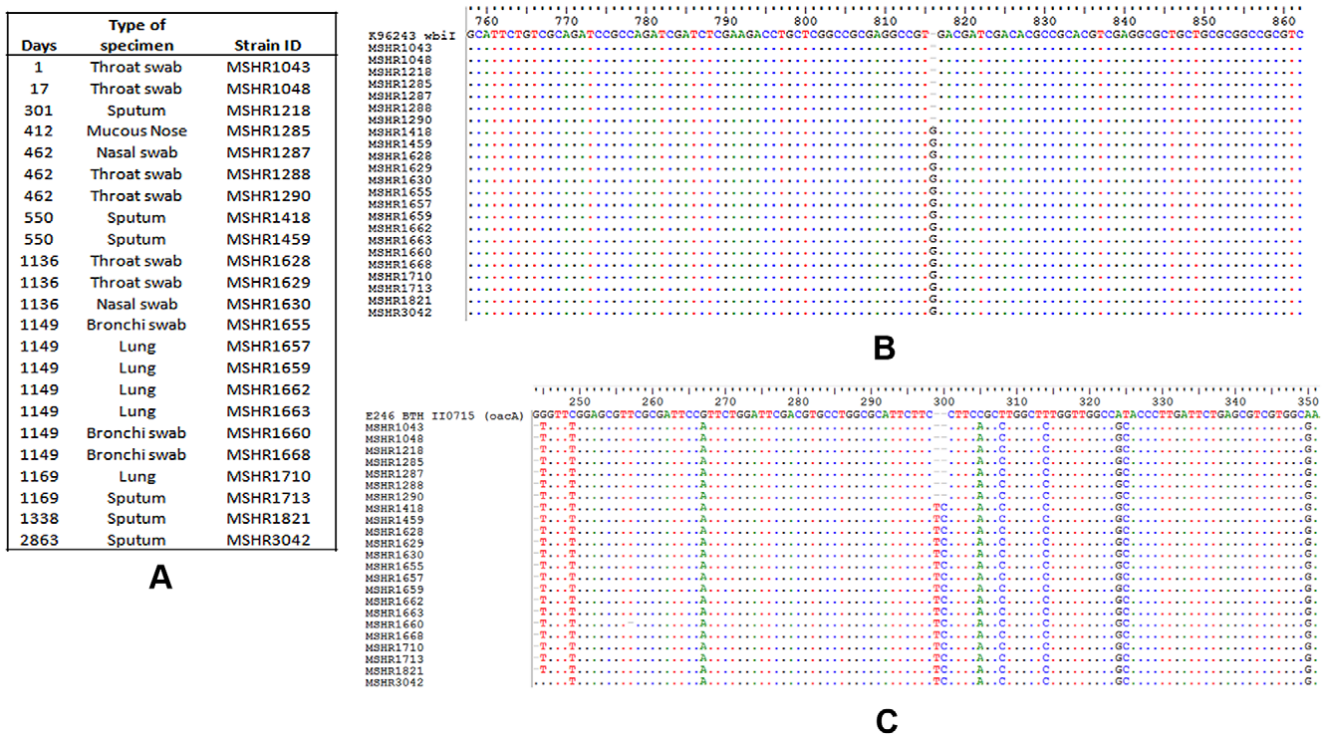
Point mutations found in *wbiI* and *oacA* genes in clonal *B. pseudomallei* strains. These strains were collected chronologically from a single chronic lung patient who had severe bronchiectasis associated with melioidosis over almost 8 years. Panel A is the chronological order of these *B. pseudomallei* strains. Panel B demonstrates an extra base (“G”) that was found to cause frame-shift mutation in *wbiI* gene of all *B. pseudomallei* strains collected from day 550 onward. Panel C demonstrates the insertion of two extra bases “TC” in BPSL1936, the *oacA* homolog, in the same strains that had the *wbiI* mutation. Note: the *wbiI* gene of *B. pseudomallei* K96243 and *oacA* gene of *B. thailandensis* E264 [14] were used as comparisons.

**Figure 5:**
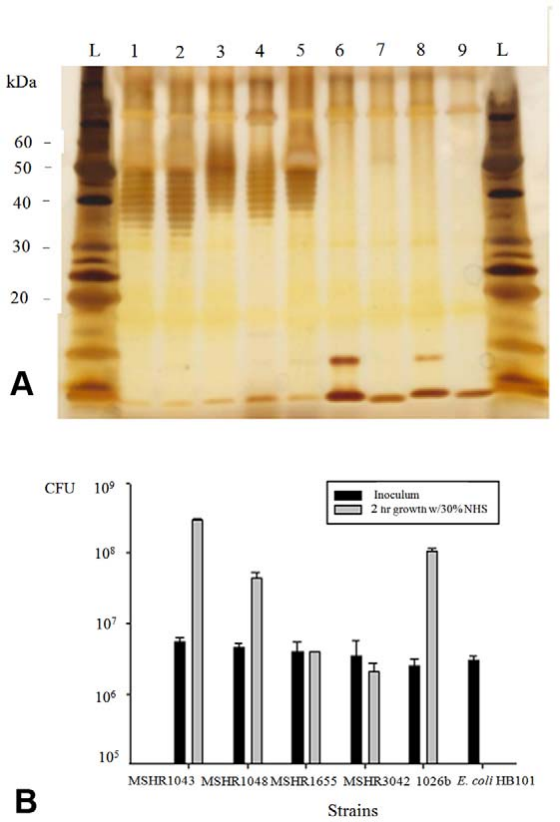
Differential LPS phenotypes and serum susceptibility of the chronic lung strains. Panel A demonstrates LPS phenotypes based upon SDS-PAGE analysis of select chronic lung strains; lanes 1–9, LPS samples from the chronic lung strains MSHR1043, MSHR1048, MSHR1218, MSHR1288, MSHR1290, MSHR1418, MSHR1459, MSHR1655, and MSHR3042, respectively; L, protein standard ladder. Panel B shows differential serum susceptibility in four select chronic lung *B. pseudomallei* strains grown in 30% of normal human serum (NHS); a well-known serum resistant *B. pseudomallei* strain 1026b, and a laboratory *E. coli* strain HB101 were used as the positive and negative controls in this study, respectively. We note that strains MSHR1655 and MSHR3042, the rough LPS strains that had mutation in their *wbiI* genes were unable to multiply in the presence of 30% NHS, whereas, the typical LPS strains MSHR1043 and MSHR1048 from the same patient were able to utilize the NHS as nutrients.

**Figure 6:**
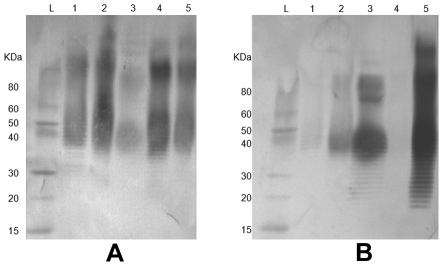
Phenotypic effects of the *oacA* mutation in *B. pseudomallei* 112 and *B. thailandensis* TXDOH revealed by immunoblot analysis. LPS samples from *B. pseudomallei* K96243 and 112, *B. mallei* ATCC23344, and *B. thailandensis* E264 and TXDOH, Lanes 1–5, respectively, hybridized against serotype A patient's serum (panel A), and *B. mallei* LPS-specific mAb 3D11 (panel B). As predicted, LPS samples from *B. pseudomallei* 112 (lane 2) and *B. thailandensis* TXDOH (lane 5) were strongly positive to the mAb 3D11 due to the mutation of their *oacA* genes. Lane L is a pre-stained protein standard ladder.

A frame-shift mutation observed in the O-antigen biosynthesis *wbiI* gene of *B. pseudomallei* strain MSHR1655 was correlated with its rough phenotype. This is one of nearly 100 strains that were isolated over 8 years from a patient with severe bronchiectasis associated with melioidosis. The mutation was an extra guanine inserted after nucleotide position 815 of the *wbiI* gene (Figure 4). The *wbiI* gene encodes an oligosaccharide epimerase/dehydratase and is conserved in all O-antigen biosynthesis gene clusters of *B. pseudomallei*. A mutation in this gene probably impacts on the synthesis of the O-antigen in this bacterial strain. There were 23 serial *B. pseudomallei* isolates observed from the chronically infected patient and the *wbiI* gene sequences were determined in all of them to detect frame shift mutations. The frame-shift mutation occurred in 16 isolates, all of which were collected on or after day 550 of the infection. The wild type sequence was present in the other seven isolates from earlier in the infection (Figure 4). Moreover, phenotypic characterization revealed that LPS samples extracted from the 16 *wbiI* mutated strains did not have the O-antigen ladder pattern (i.e the rough phenotype) based upon SDS-PAGE and silver straining (Figure 5A). Thus, it seems likely the frame-shift mutation in the *wbiI* gene blocks synthesis of the O-antigen. A recent study has reported that *oacA* gene, known to be involved in the acetylation at the *O*-4 position of the L-6dTal*p* residues of *B. thailandensis* O-antigen [14], is mutated in *B. pseudomallei* MSHR1655. Since MSHR1655 was isolated from the same patient above, we then sequenced the *oacA* gene in all of these clonal strains. We found that the *oacA* mutation occurred in the same 16 strains that had the *wbiI* mutation (Figure 4C). Additional study of the *oacA* gene in other whole genome sequenced strains determined that *B. pseudomallei* 112 and *B. thailandensis* TXDOH also had point mutation in their *oacA* genes (Table 1; Figure S1). To determine if the *oacA* gene plays only a single role in the side group modification of the L-6dTal*p* residues, or a dual role in combination with the synthesis of the O-antigen, both strains were tested for O-antigen production and immunogenic specificity. We found that *B. pseudomallei* 112 and *B. thailandensis* TXDOH expressed O-antigen type A ladder pattern and their O-antigen bands were strongly positive with the *B. mallei* LPS-specific mAb 3D11 (Figure 6) that recognized the lack of *4-O* acetylation of the L-6dTal*p* residues [14]. This suggests the *oac*A gene in *B. pseudomallei* and *B. thailandensis* has a role in the acetylation at the *O*-4 position of the O-antigen L-6dTal*p* residues but is not involved in the synthesis of the O-antigen. Thus, we determined that the rough LPS phenotype observed in the 16 clonal chronic lung strains was due to the mutation of their *wbiI* gene, but not from the effect of the *oacA* mutation. In this study, we also identified six other independent rough LPS strains, but mutations did not occur in their *wbiI* or *oacA* genes. Searching for mutations in other genes of these strains warrants a follow up study to understand alternate mechanisms that generate the rough phenotype.

Because LPS is essential for outer membrane integrity and serum resistance, four *B. pseudomallei* strains from this chronic lung patient were further tested in serum bactericidal assays. Two of the *wbiI* mutant strains that expressed the rough LPS phenotype (MSHR1655 and MSHR3042) were unable to grow in the presence of 30% normal human serum (NHS). In contrast, two early infection isolates from the same patient expressing the typical LPS A phenotype (MSHR1043 and MSHR1048) were able to resist the inhibitory human serum effect and grow (Figure 5B). Furthermore, we also confirmed that the LPS genotype B2 strains were killed in growth media containing 30% NHS, whereas the LPS genotype B strains were resistant (Figure S2). We believe that this finding of serum susceptibility in LPS genotype B2 is important and deserves further investigation.

## Discussion

### Two major (A, B) and one minor (B2) LPS genotypes exist unequally in *B. pseudomallei* populations

Despite the fact that genes responsible for the O-antigen biosynthesis in *B. pseudomallei* 1026b were identified many years ago [4], diversity of these genes across multiple *B. pseudomallei* strains has not been well studied until now. Advances in genome sequencing and comparative genomics have provided insights into the complexity and diversity of *B. pseudomallei* genomes. *B. pseudomallei* genomic studies can now strive for correlations between genomic diversity and differential phenotypes; perhaps the clinical outcomes of individual strains of *B. pseudomallei* may be predicted using basic genomic analysis. In our current study, we were able to establish a correlation between differential LPS phenotypes and diversity of O-antigen biosynthesis genes or known as LPS genotypes. Three different major LPS genotypes have been identified so far. LPS genotype A was designated to the strains that contained the O-antigen biosynthesis genes that were identical or very similar to those found in a reference strain 1026b [4], whereas the LPS genotype B category is represented by the atypical LPS strain 576. Finally, LPS B2 genotype was identified as a variant of the LPS genotype B because many of its O-antigen biosynthesis genes were similar to those of LPS genotype B, and both groups were serotype B positive. LPS genotype A was the most common genotype in both geographic locations: Southeast Asia and Australia where it accounted for 97.7% and 85.3% of the populations, respectively. Interestingly, the frequency of LPS genotype B was relatively high (approx. 13.8%) in Australian strains, whereas they accounted for only 2.3% of the strains from Southeast Asia. LPS genotype B2 was found in only 7 strains, 5 of which were from Australia, and the other 2 strains were from Papua New Guinea. In addition, LPS genotype B2 was also found in a member of *B. thailandensis*-like species which was recently discovered in Australia [22]. This would suggest that the LPS genotype B2 genes in *B. pseudomallei* may be acquired by horizontal gene transfer from a common soil bacterial species in Australia, or vice versa. Comparative genomics and phenotypic characterization of this LPS genotype B2 in *B. pseudomallei* and its near-relative species warrants further investigation.

Because the LPS genotypes B and B2 were frequently found in Australia but not in Southeast Asia, it is possible that this finding may be due to different therapies used for clinical cases in these 2 endemic locations. We have investigated this and found that the majority of these isolates were obtained before any exposure to antibiotics or treatment therapy. In addition, some of the LPS genotype B strains were collected from soil in Australia, and 2 strains of the LPS genotype B2 were found in animal cases. This confirms that the occurrence of LPS types B and B2 in Australia is not associated with the exposure to antibiotics or treatment therapy. Although, we phenotyped only 24% of the isolates that were genotyped, most tested strains were perfectly matched between their genotypes and phenotypes, except those 16 rough LPS genotype A strains from a single chronic case that had mutations in their *wbiI* genes (Figure 4). In this current study, we were unable to identify the genetic basis or mutations in 6 independent LPS genotype A strains that did not produce the O-antigen (Table S2).

Because the typical LPS was also found in *B. thailandensis*, the use of anti-LPS antibody based latex agglutination for the identification of *B. pseudomallei* in environmental specimens was not successful in an early study [23]. *B. thailandensis* LPS has also been shown to cross-react with rabbit and mouse sera obtained from inoculation with *B. pseudomallei* or *B. mallei* suggesting that LPS molecules from *B. thailandensis*, a non-pathogenic bacterium, may be useful in ongoing efforts to develop novel vaccines and/or diagnostic reagents [24]. This has brought to our attention whether low-grade *B. thailandensis* infections might naturally provide protection against melioidosis. Although the O-antigen biosynthesis genes in *B. pseudomallei* and *B. thailandensis* are similar, a recent study by a Singaporean group has revealed that lipid A components of the LPS from both *B. pseudomallei* and *B. thailandensis* must be different; the murine and human macrophages produced lower levels of tumor necrosis factor alpha, interleukin-6 (IL-6), and IL-10 in response to *B. pseudomallei* LPS than in response to *B. thailandensis* LPS *in vitro* [25]. In our current study, the typical LPS was also found in *B. oklahomensis* strain EO147, formerly known as an American *B. pseudomallei* strain [26], suggesting that the typical LPS is widely spread in multiple *Burkholderia* species. This group includes highly pathogenic species such as *B. pseudomallei* and *B. mallei*, but also non-pathogenic species: *B. thailandensis*, *B. thailandensis*-like species, and *B. oklahomensis*. The evolution of LPS diversity across these closely related species is likely a function of differential selection and horizontal transfer of genetic elements. This diversity could play a role in frequency and distribution of disease in humans. However, without understanding molecular structures of these O-antigen types, it is difficult to access the phenotypic effects of this genetic diversity. Structural analysis of the O-antigen types B and B2 deserves further investigations. In addition, we have found that the LPS genotype B2 strains were sensitive to 30% NHS, whereas the LPS type B strains were resistant (Figure S2). This finding demonstrates a level of phenotypic differences between these two serologically related groups. We believe that the consequences for case treatment associated with these differential serum susceptibilities also warrant further investigations.

### The rough LPS phenotype – Adaptation to survival and persistence in a host?

A previous study has shown that the two less common LPS phenotypes (smooth type B and rough type) were more prevalent in clinical than environmental isolates and more prevalent in Australian isolates than Thai isolates [10]. In our current study, LPS genotype B was found in both clinical and environmental strains from Australia, whereas the rough LPS was still found only in clinical strains. Based on our description of the molecular basis for LPS phenotypes, it is unlikely that *B. pseudomallei* will readily switch its LPS phenotype from A to B, or vice versa, as has been suggested previously [10]. The gene compositions of LPS genotypes A and B are very different and a simple switching mechanism is difficult to envision. In addition, we have found that at least some rough LPS strains have mutations in their O-antigen biosynthesis genes. These include 16 clonally related isolates from a single chronic lung infected patient (Table S2). All of these strains were identified as LPS genotype A with mutations in their O-antigen biosynthesis genes. Using Tn5-OT182 mutagenesis, DeShazer and colleagues identified at least seven genes in the O-antigen biosynthesis operon of *B. pseudomallei* 1026b that were responsible for O-antigen biosynthesis and serum resistance; these included *rmlB*, *rmlD*, *wbiA*, *wbiC*, *wbiE*, *wbiG*, and *wbiI* [4]. In our current study, we found point mutations in *wbiI* and *oacA* genes of *B. pseudomallei* isolates that were collected from a chronic lung patient (Figure 4). We hypothesize that the frame-shift mutation in the *wbiI* genes blocks O-antigen biosynthesis in all mutant strains, but not from the effect of the *oacA* mutation. This is because we observed the *oacA* mutations in *B. pseudomallei* 112 and *B. thailandensis* TXDOH that had normal O-antigen biosynthesis gene cluster (Table 1 and Figure S1). Our study has demonstrated that these two *oacA* mutant strains expressed O-antigens identical to those found in *B. mallei* due to lack of the 4-*O* acetylation of the L-6dTal*p* residues of the O-antigen. The lack of the 4-*O* acetylation of the L-6dTal*p* residues has recently been described in the *oacA* knock-out mutant *B. thailandensis* ZT0715 and a wild-type *B. mallei* ATCC23344 [14].

We have demonstrated that these *wbiI* mutant strains produced rough LPS and were sensitive to normal human serum suggesting that the *wbiI* gene encoding for epimerase, or dehydratase, was essential for the biosynthesis of *B. pseudomallei* O-antigen. Although loss of the O-antigen might compromise serum survival it might also be adaptive in particular niches. *B. pseudomallei* survival or persistence in the host might be enhanced without the surface presentation of the O-antigenic moiety of the LPS, as it would not be recognized by host immune systems and would, therefore, avoid being killed by antibodies. The O-antigenic polysaccharide of *B. pseudomallei* modulates the host cell response, which in turn controls the intracellular fate of *B. pseudomallei* inside macrophage. This was concluded from the observation that the O-antigen mutant *B. pseudomallei* strain SRM117 was more susceptible to macrophage killing during the early phase of infection than the parental wild-type strain 1026b [27]. This was also confirmed by the same group when they demonstrated the importance of intracellular killing by the human polymorphonuclear cells (PMNs), macrophages (Mϕs), and susceptibility to killing by 30% normal human serum [28].

LPS and CPS (capsular polysaccharide) have been used as subunits in immunizing BALB/c mice against *B. pseudomallei* infection [2]. Mice vaccinated with LPS developed predominantly IgM and IgG3 responses, whereas the mice vaccinated with the CPS developed a predominantly IgG2b response. Furthermore, immunization with the LPS provided an optimal protective response, and the immunized mice challenged by the aerosol route showed a small increase in the mean time to death compared with the unvaccinated controls [2]. Previously, it was shown that *B. pseudomallei* LPS from strain 1026b signaled through Toll-like receptor (TLR) 2 and not through TLR4 [29]. This was observed in the TLR2 knock-out mutant mice that displayed a markedly improved host defense, but it was not observed in TLR4 knock-out mice [29]. In contrast, a study in HEK293 cells demonstrated that heat-killed *B. pseudomallei* strains K96243 or BP-1 activated TLR2 and TLR4, and in the presence of MD-2, LPS and lipid A from BP-1 are TLR4 ligands [30]. We note that *B. pseudomallei* 1026b and K96243 expressed the typical O-antigen type A, but the O-antigen type of BP-1 was not reported in that study. Although there was no report of association between the LPS types and disease severity (e.g., fatal versus non-fatal, and septicemia versus localized), clinical manifestations (neurologic versus non-neurologic), or underlying risk factors (diabetic versus non-diabetic) observed in a previous study [10], full phenotypic characterization including virulence in animal models, innate immune response, etc of these different LPS types warrants further investigations given the LPS diversity that we have described.

## Supporting Information

Figure S1 **Point mutations found in gene BPSL1936 (** ***oacA*** **homolog) of** ***B. pseudomallei*** **MSHR1655 and 112, and** ***B. thailandensis*** **TXDOH.** These point mutations (panel A): in strain MSHR1655, the mutation was associated with 2 extra bases, “TC”, inserted right after nucleotide no. 298 of this gene; in strain 112, it was associated with a deletion of one base, “T”, at nucleotide no. 112; and in *B. thailandensis* TXDOH, it was associated with the 5′ truncation mutation. Amino acid sequence analysis (panel B) has demonstrated that the point mutations in MSHR1655 and 112 potentially caused frame-shift mutations in their BPSL1936 genes, and then split the gene into 2 separated open reading frames (ORFs). Two known amino acid motifs, VXXFFXXSG and WXLXXEXXXY, were present in both ORFs of MSHR1655, whereas only the latter motif was present in strain 112. We noted that both amino acid motifs were absent in *B. thailandensis* TXDOH.
(PPT)

Figure S2 **O-antigen types and differential serum susceptibility.** O-antigen type A and B strains including *B. pseudomallei* 1026b, NCTC13178, and NCTC13179, MSHR367b, MSHR98, respectively, were resistant to 30% normal human serum (NHS); whereas the O-antigen type B2 strains : *B. pseudomallei* MSHR840, MSHR454, and MSHR1950, and *B. thailandensis-like sp*. strain MSMB121 were sensitive to the 30% NHS. A rough O-antigen type strain MSHR3042, a member of the chronic lung strains (see text), was also sensitive. We noted that *B. thailandensis* E264 was able to survive, but unable to multiple in the presence of 30% NHS. *E. coli* HB101 was used as a control serum sensitive strain.
(PPT)

Table S1 **Comparison of LPS genotype A, B, and B2 gene clusters.**
(DOC)

Table S2 **List of bacterial strains used in this study and their LPS genotyping PCR results.**
(XLS)
