# Supplementary figures and images for: The Genetic and Molecular Basis of O-Antigenic Diversity in Burkholderia pseudomallei Lipopolysaccharide
Source: PLoS Negl Trop Dis. 2012 Jan 3;6(1):e1453. doi: 10.1371/journal.pntd.0001453 (PMC3250505; doi:10.1371/journal.pntd.0001453)

## Slide 1
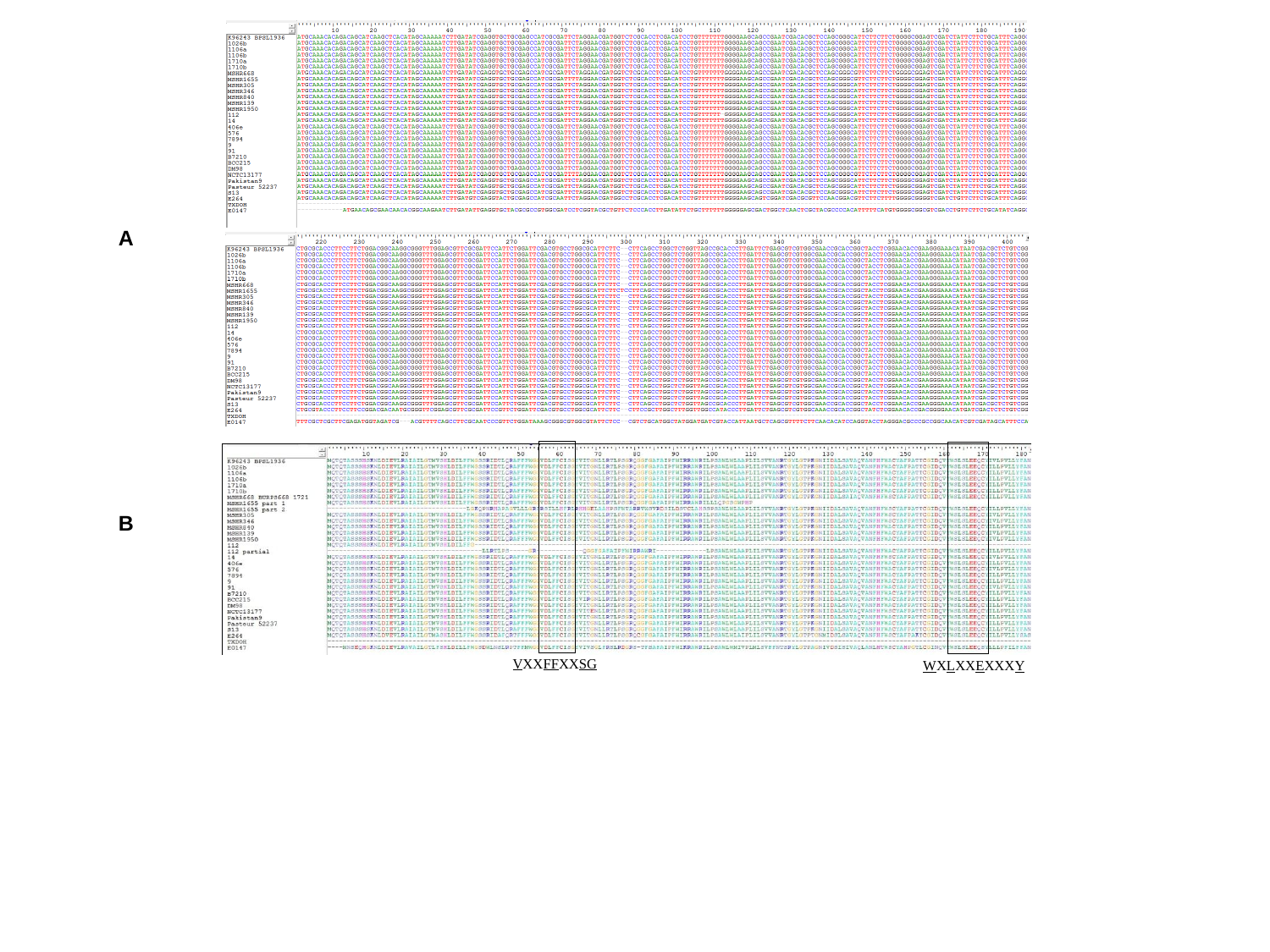

A
B
VXXFFXXSG
WXLXXEXXXY

Supplement: Figure S1 — Point mutations found in gene BPSL1936 ( oacA homolog) of B. pseudomallei MSHR1655 and 112, and B. thailandensis TXDOH. These point mutations (panel A): in strain MSHR1655, the mutation was associated with 2 extra bases, “TC”, inserted right after nucleotide no. 298 of this gene; in strain 112, it was associated with a deletion of one base, “T”, at nucleotide no. 112; and in B. thailandensis TXDOH, it was associated with the 5′ truncation mutation. Amino acid sequence analysis (panel B) has demonstrated that the point mutations in MSHR1655 and 112 potentially caused frame-shift mutations in their BPSL1936 genes, and then split the gene into 2 separated open reading frames (ORFs). Two known amino acid motifs, VXXFFXXSG and WXLXXEXXXY, were present in both ORFs of MSHR1655, whereas only the latter motif was present in strain 112. We noted that both amino acid motifs were absent in B. thailandensis TXDOH. (PPT) [file pntd.0001453.s001.ppt]
