# Supplementary material for: The Genetic and Molecular Basis of O-Antigenic Diversity in Burkholderia pseudomallei Lipopolysaccharide
Source: PLoS Negl Trop Dis. 2012 Jan 3;6(1):e1453. doi: 10.1371/journal.pntd.0001453 (PMC3250505; doi:10.1371/journal.pntd.0001453)
Supplement: Figure S2 — O-antigen types and differential serum susceptibility. O-antigen type A and B strains including B. pseudomallei 1026b, NCTC13178, and NCTC13179, MSHR367b, MSHR98, respectively, were resistant to 30% normal human serum (NHS); whereas the O-antigen type B2 strains : B. pseudomallei MSHR840, MSHR454, and MSHR1950, and B. thailandensis-like sp. strain MSMB121 were sensitive to the 30% NHS. A rough O-antigen type strain MSHR3042, a member of the chronic lung strains (see text), was also sensitive. We noted that B. thailandensis E264 was able to survive, but unable to multiple in the presence of 30% NHS. E. coli HB101 was used as a control serum sensitive strain. (PPT) [file pntd.0001453.s002.ppt]

## Slide 1
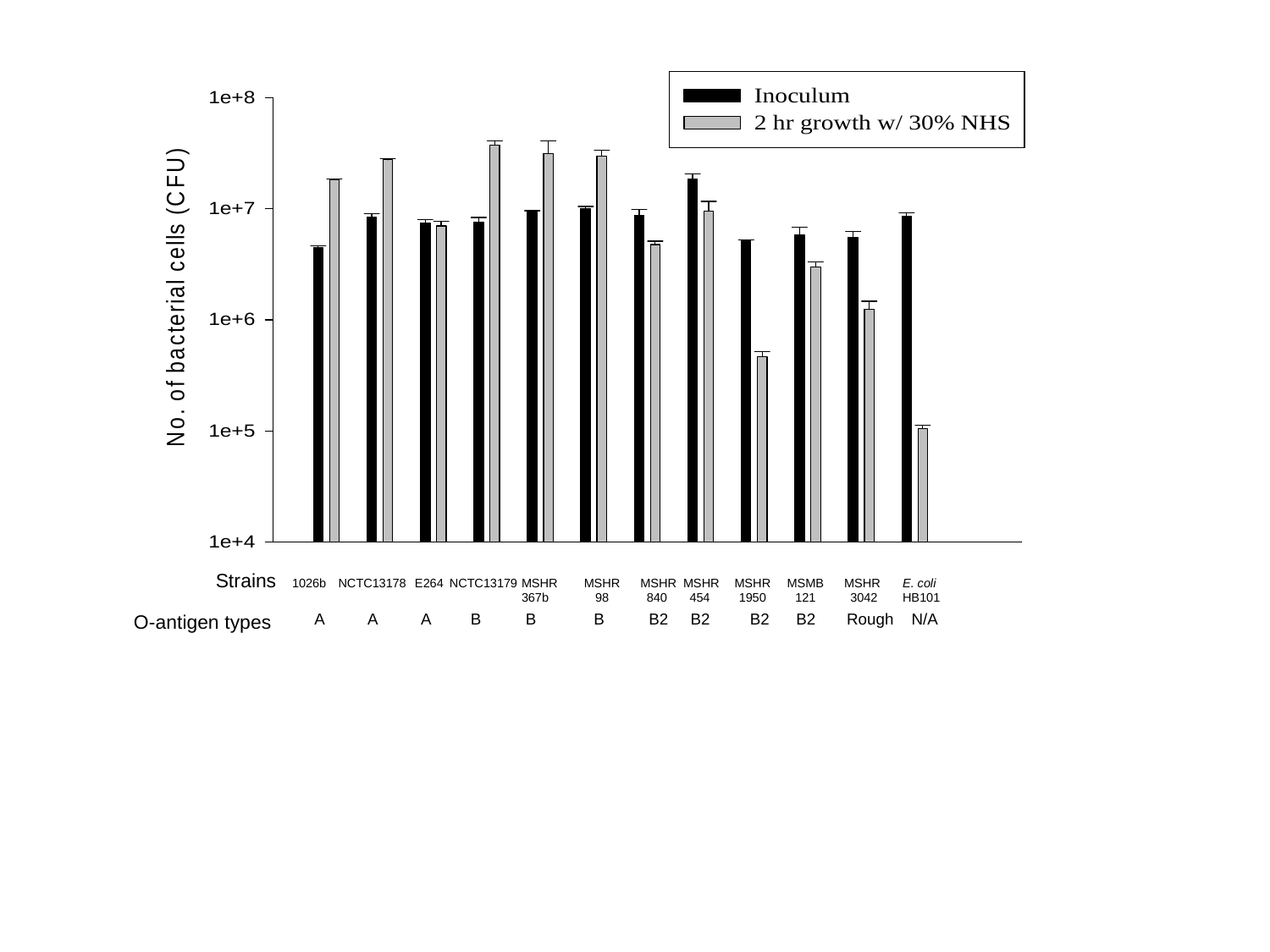

Strains
1026b
NCTC13178
E264
NCTC13179
MSHR 367b
MSHR 98
MSHR 840
MSHR 454
MSHR 1950
MSMB 121
MSHR 3042
E. coli HB101
O-antigen types
 A A A B B B B2 B2 B2 B2 Rough N/A
