# Supplementary material for: The Genetic and Molecular Basis of O-Antigenic Diversity in Burkholderia pseudomallei Lipopolysaccharide
Source: PLoS Negl Trop Dis. 2012 Jan 3;6(1):e1453. doi: 10.1371/journal.pntd.0001453 (PMC3250505; doi:10.1371/journal.pntd.0001453)
Supplement: Table S1 — Comparison of LPS genotype A, B, and B2 gene clusters. (DOC) [file pntd.0001453.s003.doc]

Table S1. Comparison of LPS genotype A, B, and B2 gene clusters.

|  |  | **Type A** | | **Type B** | | **Type B2** | | **Note** |
| --- | --- | --- | --- | --- | --- | --- | --- | --- |
| **Gene** | **Function** | **Gene Name** | **Locus Tag** | **Gene Name** | **Locus Tag** | **Gene Name** | **Locus Tag** |  |
| *wbiI* | Putative epimerase/dehydratase | *wbiI* | BPSL2672 | *wbiI* | BUC_3392 | *wbiI* | BURP840_LPSb01 | Highly similar |
| *wbiH* | N-acetylglucosaminyltransferase | *wbiH* | BPSL2673 | *wbiH* | BUC_3393 | *wbiH* | BURP840_LPSb02 | Highly similar |
| *wbiG* | Putative epimerase/dehydratase | *wbiG* | BPSL2674 | *wbiG* | BUC_3394 | *wbiG* | BURP840_LPSb03 | Highly similar |
| *wbiF* | Glycosyl transferase | *wbiF* | BPSL2675 | Unassigned | BUC_3395 | Unassigned | BURP840_LPSb04 | Varied |
| *wbiE* | Glycosyl transferase | *wbiE* | BPSL2676 | *-* | *-* | - | *-* | Unique |
| *wbiD* | O-antigen methyl transferase | *wbiD* | BPSL2677 | *-* | *-* | - | *-* | Unique |
| *wbiC* | Glycosyl transferase | *wbiC* | BPSL2678 | *-* | *-* | - | *-* | Unique |
| *wbiB* | Putative epimerase/dehydratase | *wbiB* | BPSL2679 | *-* | *-* | - | *-* | Unique |
| *wbiA* | Putative O-antigen acetylase | *wbiA* | BPSL2680 | *-* | *-* | - | *-* | Unique |
| *wzt* | ABC transporter, ATP-binding protein | *wzt* | BPSL2681 | Unassigned | BUC_3406 | Unassigned | BURP840_LPSb09 | Varied |
| *wzm* | Putative ABC transporter, membrane permease | *wzm* | BPSL2682 | *-* | - | - | - | Unique |
| *rmlD* | dTDP-4-dehydrorhamnose reductase | *rmlD* | BPSL2683 | *-* | - | *-* | - | Unique |
| *rmlC* | dTDP-6-deoxy-D-glucose-3,5 epimerase | *rmlC* | BPSL2684 | *rfbC* | BUC_3413 | *rmlC* | BURP840_LPSb19 | Highly similar |
| *rmlA* | Glucose-1-phosphate thymidylyltransferase | *rmlA* | BPSL2685 | *rfbA* | BUC_3414 | *rmlA* | BURP840_LPSb19a | Highly similar |
| *rmlB* | dTDP-glucose 4,6-dehydratase | *rmlB* | BPSL2686 | *rfbB* | BUC_3415 | *rmlB* | BURP840_LPSb20 | Highly similar |
| BUC_3396 | Putative membrane protein | *-* | *-* | Unassigned | BUC_3396 | - | - | Unique |
| BUC_3397 | Putative acyltransferase | *-* | *-* | Unassigned | BUC_3397 | Unassigned | BURP840_LPSb05 | Similar |
| BUC_3398 | Integral membrane protein | *-* | *-* | Unassigned | BUC_3398 | - | - | Unique |
| BUC_3399 | FAD linked oxidase domain protein | *-* | *-* | Unassigned | BUC_3399 | - | - | Unique |
| BUC_3400 | Dehydrogenase | *-* | *-* | Unassigned | BUC_3400 | - | - | Unique |
| BUC_3401 | Putative membrane protein | *-* | *-* | Unassigned | BUC_3401 | - | - | Unique |
| BUC_3402 | NAD-dependent epimerase/dehydratase | *-* | *-* | Unassigned | BUC_3402 | - | - | Unique |
| BUC_3403 | Amine oxidase | *-* | *-* | Unassigned | BUC_3403 | - | - | Unique |
| BUC_3404 | GtrA family protein | *-* | *-* | Unassigned | BUC_3404 | - | - | Unique |
| BUC_3405 | Group 1 glycosyl transferase | *-* | *-* | Unassigned | BUC_3405 | Unassigned | BURP840_LPSb06-08 | Varied |
| BUC_3407 | Sulfotransferase domain protein | *-* | *-* | Unassigned | BUC_3407 | Unassigned | BURP840_LPSb10 | Varied |
| BUC_3408 | ABC-2 type transporter | *-* | *-* | Unassigned | BUC_3408 | Unassigned | BURP840_LPSb11 | Similar |
| BUC_3409 | TagH teichoic acids export ATP-binding protein | *-* | *-* | Unassigned | BUC_3409 | Unassigned | BURP840_LPSb12 | Similar |
| BUC_3410 | Family 2 glycosyl transferase | *-* | *-* | Unassigned | BUC_3410 | - | - | Unique |
| BUC_3411 | Rhamnosyltransferase | *-* | *-* | Unassigned | BUC_3411 | - | - | Unique |
| BUC_3412 | dTDP-4-dehydrorhamnose reductase | *-* | *-* | *rfbD* | BUC_3412 | Unassigned | BURP840_LPSb18 | Varied |
| BURP840_LPSb13 | Putative D-α,β-D-heptose 1,7 bisphosphate phosphatase | *-* | *-* | - | - | Unassigned | BURP840_LPSb13 | Unique |
| BURP840_LPSb14 | Putative nucleotidyl transferase | *-* | *-* | - | - | Unassigned | BURP840_LPSb14 | Unique |
| BURP840_LPSb15 | Putative phosphoheptose isomerase | *-* | *-* | - | - | Unassigned | BURP840_LPSb15 | Unique |
| BURP840_LPSb16 | Putative GHMP kinase | *-* | *-* | - | - | Unassigned | BURP840_LPSb16 | Unique |
| BURP840_LPSb17 | Putative glycosyl transferase | *-* | *-* | - | - | Unassigned | BURP840_LPSb17 | Unique |

Note: A (-) indicates no homologous gene within that LPS type.
